# Supplementary material for: Dual Topoisomerase Inhibitor Is Highly Potent and Improves Antitumor Response to Radiotherapy in Cervical Carcinoma
Source: Int J Mol Sci. 2025 Mar 21;26(7):2829. doi: 10.3390/ijms26072829 (PMC11988843; doi:10.3390/ijms26072829)
Supplement: Supplementary file 1 [file ijms-26-02829-s001.zip › Supplementary Flies/250304_SI.pdf]

International Journal of *Molecular Sciences*

MDPI

Supplementary Materials

Dual topoisomerase inhibitor is highly potent and improves antitumor response to radiotherapy in cervical carcinoma

Inken Flörkemeier [1,2,10,*], Hannah L. Hotze [1], Anna Lena Heyne[1,2,], Jonas Hildebrandt [2], Jörg P. Weimer [1], Nina Hedemann [1], Christoph Rogmans [1], David Holthaus [1], Frank-Andre Siebert [3], Markus Hirt [3], Robert Polten [4], Michael Morgan [4], Rüdiger Klapdor [5,6], Axel Schambach [4,7], Astrid Dempfle [8], Nicolai Maass [1], Marion T. van Mackelenbergh [1], Bernd Clement [2] and Dirk O. Bauerschlag [1,9]

Supplementary Materials

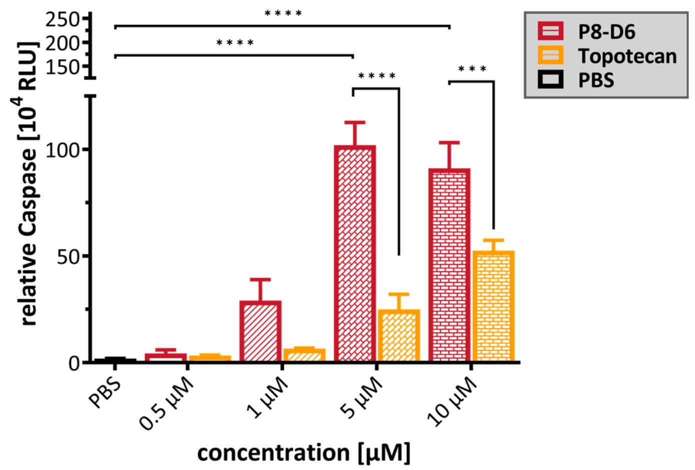

Figure S 1 Apoptosis induction of P8-D6 and topotecan treatment in SiHa cells. SiHa cells were treated in a 2D monolayer cell culture with different concentrations of P8-D6, topotecan and PBS in single therapy for 48 h. Subsequently, the viability and caspase activity were determined. The apoptosis rate is represented by the relative caspase activity (relative luminescence units, RLU). Data are means + SEM one-way ANOVA, *** (p <0.001), **** (p <0.0001).

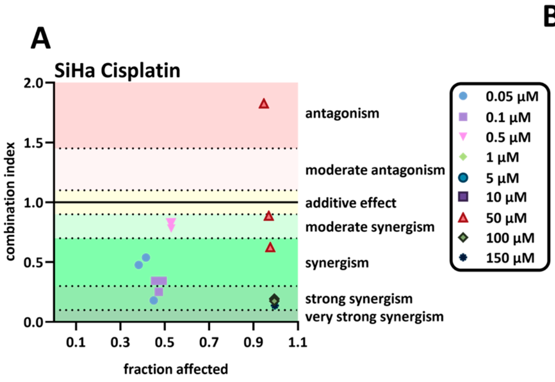

B

combination therapy cisplatin [µM]/	radiation [Gy]	fa	monotherapy cisplatin [µM]	monotherapy radiation [Gy]	dose reduction index cisplatin	dose reduction index radiation
0.05	2.5	0.450	0.43	39.14	8.63	15.66
0.1	2.5	0.473	0.49	52.41	4.90	20.97
0.5	2.5	0.529	0.66	104.6	1.32	41.85
1	2.5	0.456	0.45	42.51	0.45	17.00
5	2.5	0.580	0.87	200.2	0.17	80.07
10	2.5	0.609	1.03	291.2	0.10	116.5
50	2.5	0.969	56.34	3.09 x10^6	1.13	1.24 x10^6
100	2.5	0.994	526.5	5.46 x10^8	5.26	2.18 x10^8
150	2.5	0.995	766.0	1.30 x10^9	5.11	5.20 x10^8
0.05	5	0.382	0.30	16.29	5.91	3.26
0.1	5	0.456	0.45	42.05	4.45	8.41
0.5	5	0.523	0.64	97.60	1.28	19.52
1	5	0.462	0.46	45.27	0.46	9.05
5	5	0.541	0.71	122.3	0.14	24.46
10	5	0.574	0.84	184.7	0.08	36.95
50	5	0.947	27.38	5.82 x10^5	0.55	1.16 x10^5
100	5	0.994	511.7	5.11 x10^8	5.12	1.02 x10^8
150	5	0.996	849.2	1.65 x10^9	5.66	3.30 x10^8
0.05	10	0.415	0.36	25.14	7.13	2.51
0.1	10	0.490	0.53	64.16	5.34	6.42
0.5	10	0.533	0.67	109.8	1.35	10.98
1	10	0.435	0.40	32.25	0.40	3.23
5	10	0.495	0.55	68.85	0.11	6.89
10	10	0.628	1.14	370.2	0.11	37.02
50	10	0.976	80.11	6.99 x10^6	1.60	6.99 x10^5
100	10	0.994	572.9	6.64 x10^8	5.73	6.64 x10^7
150	10	0.996	1084.3	2.91 x10^9	7.23	2.91 x10^8

Dose reduction	DRI >10
	DRI >5
	DRI >1
Dose increase	DRI < 1

Figure S 2 Combination index (CI) analysis of radiotherapy in combination with cisplatin in SiHa spheroids. (A) Combination index (CI) for combining radiotherapy with cisplatin was determined using SiHa spheroids. CI values was calculated by CompuSyn software using the fraction affected (fa)-value. The fa-value represents the fraction of cell viability affected by therapy. fa color scalar (0 (white)-1 (light brown)). Combinations were considered synergistic when CIs were below 1.0. (B)

The monotherapy column defines the concentrations that are needed in monotherapy to affect a certain fraction of cells by therapy. The increasing blue color intensity classifies the concentration required to achieve the same effect. The more treatment required, the more intense the blue color. DRI values represent the order of magnitude (fold) of dose reduction in combination setting compared with each drug alone.

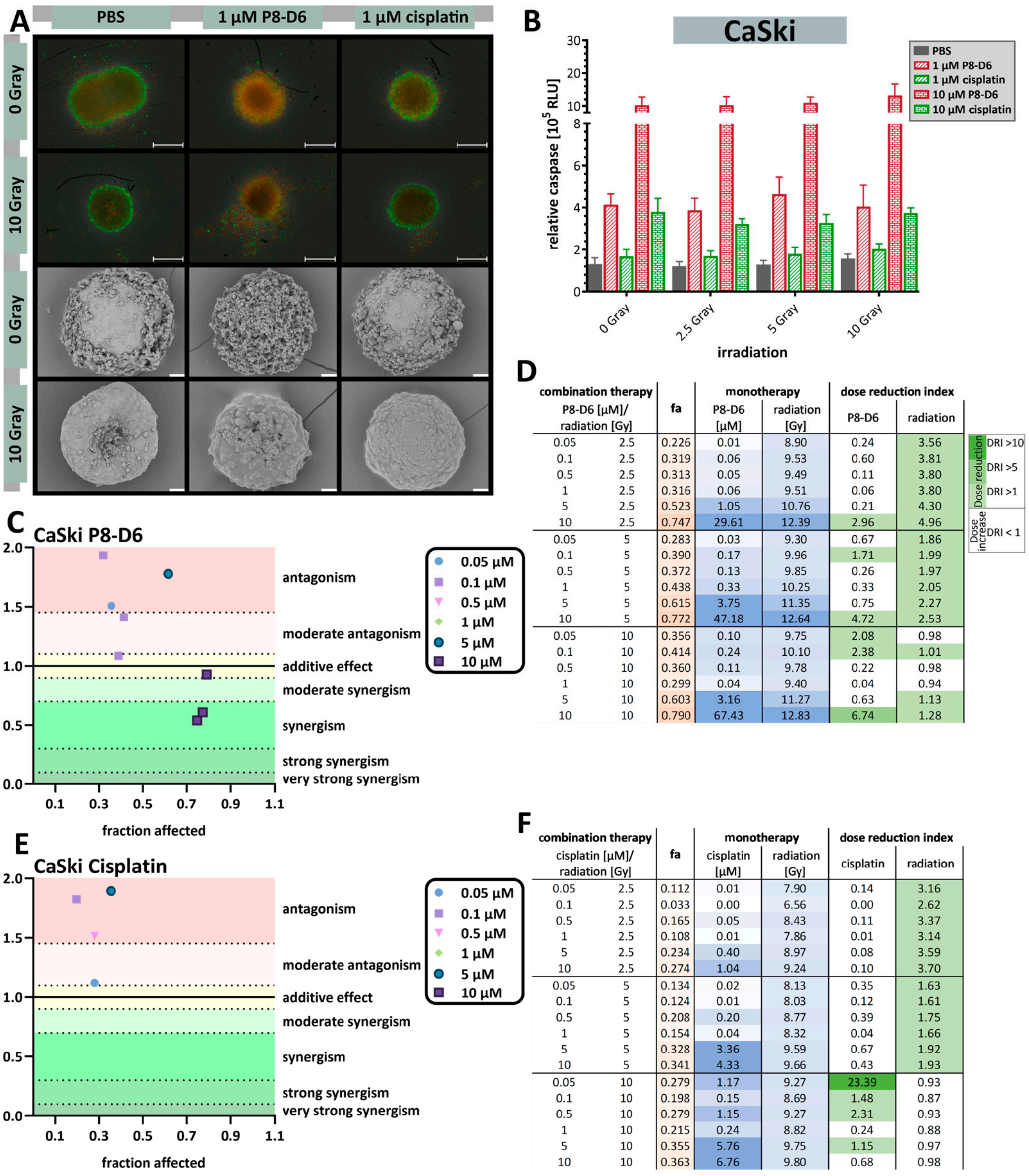

D

combination therapy P8-D6 [µM]/	radiation [Gy]	fa	monotherapy P8-D6 [µM]	monotherapy radiation [Gy]	dose reduction index P8-D6	dose reduction index radiation
0.05	2.5	0.226	0.01	8.90	0.24	3.56
0.1	2.5	0.319	0.06	9.53	0.60	3.81
0.5	2.5	0.313	0.05	9.49	0.11	3.80
1	2.5	0.316	0.06	9.51	0.06	3.80
5	2.5	0.523	1.05	10.76	0.21	4.30
10	2.5	0.747	29.61	12.39	2.96	4.96
0.05	5	0.283	0.03	9.30	0.67	1.86
0.1	5	0.390	0.17	9.96	1.71	1.99
0.5	5	0.372	0.13	9.85	0.26	1.97
1	5	0.438	0.33	10.25	0.33	2.05
5	5	0.615	3.75	11.35	0.75	2.27
10	5	0.772	47.18	12.64	4.72	2.53
0.05	10	0.356	0.10	9.75	2.08	0.98
0.1	10	0.414	0.24	10.10	2.38	1.01
0.5	10	0.360	0.11	9.78	0.22	0.98
1	10	0.299	0.04	9.40	0.04	0.94
5	10	0.603	3.16	11.27	0.63	1.13
10	10	0.790	67.43	12.83	6.74	1.28

F

combination therapy cisplatin [µM]/	radiation [Gy]	fa	monotherapy cisplatin [µM]	monotherapy radiation [Gy]	dose reduction index cisplatin	dose reduction index radiation
0.05	2.5	0.112	0.01	7.90	0.14	3.16
0.1	2.5	0.033	0.00	6.56	0.00	2.62
0.5	2.5	0.165	0.05	8.43	0.11	3.37
1	2.5	0.108	0.01	7.86	0.01	3.14
5	2.5	0.234	0.40	8.97	0.08	3.59
10	2.5	0.274	1.04	9.24	0.10	3.70
0.05	5	0.134	0.02	8.13	0.35	1.63
0.1	5	0.124	0.01	8.03	0.12	1.61
0.5	5	0.208	0.20	8.77	0.39	1.75
1	5	0.154	0.04	8.32	0.04	1.66
5	5	0.328	3.36	9.59	0.67	1.92
10	5	0.341	4.33	9.66	0.43	1.93
0.05	10	0.279	1.17	9.27	23.39	0.93
0.1	10	0.198	0.15	8.69	1.48	0.87
0.5	10	0.279	1.15	9.27	2.31	0.93
1	10	0.215	0.24	8.82	0.24	0.88
5	10	0.355	5.76	9.75	1.15	0.97
10	10	0.363	6.76	9.80	0.68	0.98

Figure S 3 Antitumor responses to combined radiotherapy and chemotherapy in CaSki spheroids. CaSki spheroids were maintained in ultra-low attachment plates for 24 h, irradiated with a total dose of 2.5 Gy, 5 Gy or 10 Gy and immediately treated for 48 h with P8-D6, cisplatin or PBS. (A top) After treatment, CaSki spheroids were live/dead cell co-stained with PI (red), calcein-AM (green) and imaged by fluorescence microscopy; scale bars, 500 µm. (A bottom) Scanning electron microscope images were taken of spheroids treated with radio-chemotherapy; scale bars, 50µm. (B) Viability and caspase activity of CaSki spheroids were measured after treatment, (relative luminescence units, RLU). (C, E) Combination index (CI) for combining radiotherapy with P8-D6 (C) or cisplatin (E) was determined using CaSki spheroids. CI values was calculated by CompuSyn software using the fraction affected (fa)-value. The fa-value represents the fraction of cell viability affected by therapy. fa color scalar (0 (white)-1 (light brown)). Combinations were considered synergistic when CIs were below 1.0. (D, F) The monotherapy column defines the concentrations that are

needed in monotherapy to affect a certain fraction of cells by therapy with P8-D6 (D) or cisplatin (F) and radiotherapy. The increasing blue color intensity classifies the concentration required to achieve the same effect. The more treatment required, the more intense the blue color. DRI values represents the order of magnitude (fold) of dose reduction in combination setting compared with each drug alone.

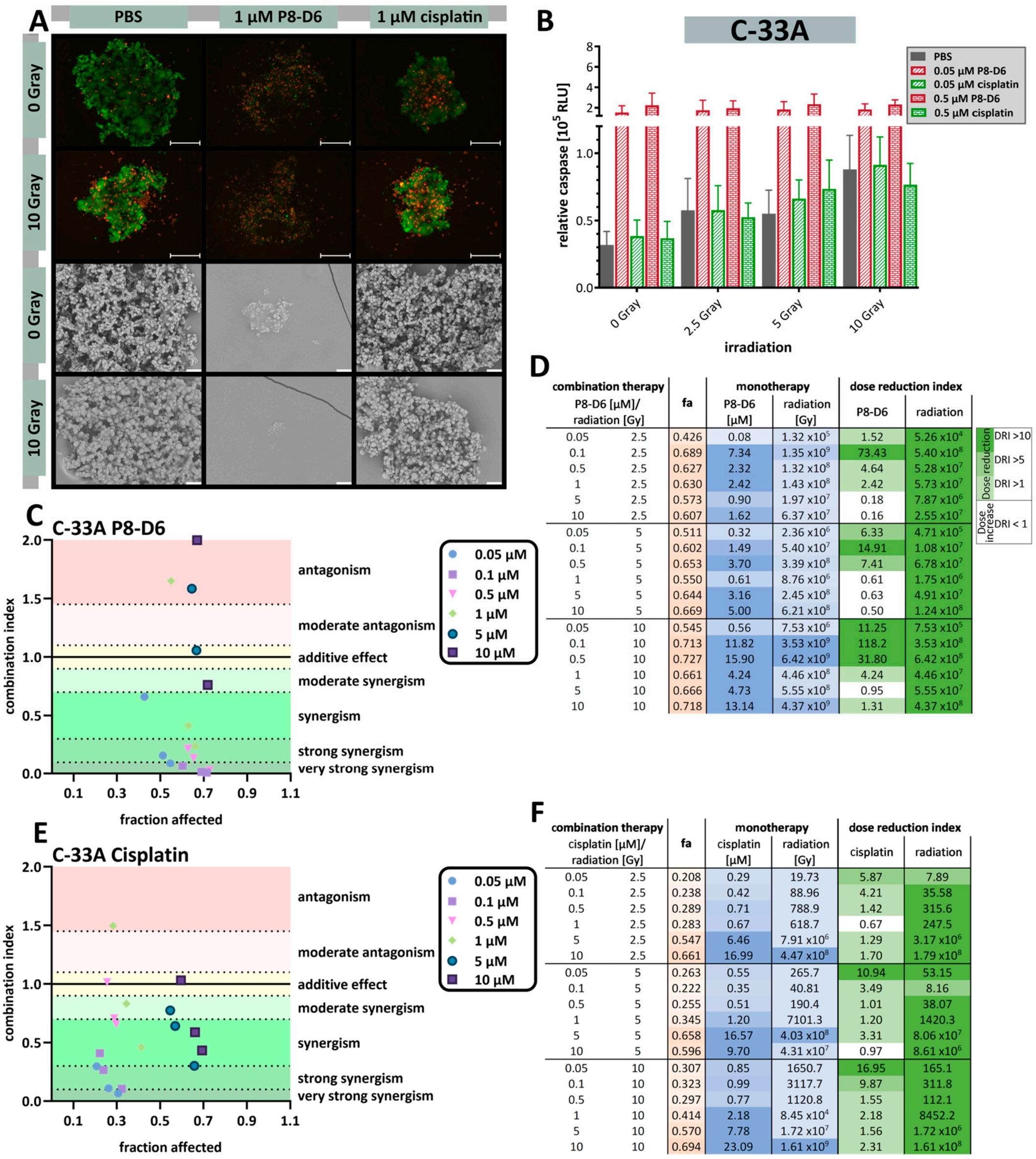

D

combination therapy P8-D6 [µM]/	radiation [Gy]	fa	monotherapy P8-D6 [µM]	monotherapy radiation [Gy]	dose reduction index P8-D6	dose reduction index radiation
0.05	2.5	0.426	0.08	1.32×10^5	1.52	5.26×10^4
0.1	2.5	0.689	7.34	1.35×10^9	73.43	5.40×10^8
0.5	2.5	0.627	2.32	1.32×10^8	4.64	5.28×10^7
1	2.5	0.630	2.42	1.43×10^8	2.42	5.73×10^7
5	2.5	0.573	0.90	1.97×10^7	0.18	7.87×10^6
10	2.5	0.607	1.62	6.37×10^7	0.16	2.55×10^7
0.05	5	0.511	0.32	2.36×10^6	6.33	4.71×10^5
0.1	5	0.602	1.49	5.40×10^7	14.91	1.08×10^7
0.5	5	0.653	3.70	3.39×10^8	7.41	6.78×10^7
1	5	0.550	0.61	8.76×10^6	0.61	1.75×10^6
5	5	0.644	3.16	2.45×10^8	0.63	4.91×10^7
10	5	0.669	5.00	6.21×10^8	0.50	1.24×10^8
0.05	10	0.545	0.56	7.53×10^6	11.25	7.53×10^5
0.1	10	0.713	11.82	3.53×10^9	118.2	3.53×10^8
0.5	10	0.727	15.90	6.42×10^9	31.80	6.42×10^8
1	10	0.661	4.24	4.46×10^8	4.24	4.46×10^7
5	10	0.666	4.73	5.55×10^8	0.95	5.55×10^7
10	10	0.718	13.14	4.37×10^9	1.31	4.37×10^8

F

combination therapy cisplatin [µM]/	radiation [Gy]	fa	monotherapy cisplatin [µM]	monotherapy radiation [Gy]	dose reduction index cisplatin	dose reduction index radiation
0.05	2.5	0.208	0.29	19.73	5.87	7.89
0.1	2.5	0.238	0.42	88.96	4.21	35.58
0.5	2.5	0.289	0.71	788.9	1.42	315.6
1	2.5	0.283	0.67	618.7	0.67	247.5
5	2.5	0.547	6.46	7.91×10^6	1.29	3.17×10^6
10	2.5	0.661	16.99	4.47×10^8	1.70	1.79×10^8
0.05	5	0.263	0.55	265.7	10.94	53.15
0.1	5	0.222	0.35	40.81	3.49	8.16
0.5	5	0.255	0.51	190.4	1.01	38.07
1	5	0.345	1.20	7101.3	1.20	1420.3
5	5	0.658	16.57	4.03×10^8	3.31	8.06×10^7
10	5	0.596	9.70	4.31×10^7	0.97	8.61×10^6
0.05	10	0.307	0.85	1650.7	16.95	165.1
0.1	10	0.323	0.99	3117.7	9.87	311.8
0.5	10	0.297	0.77	1120.8	1.55	112.1
1	10	0.414	2.18	8.45×10^4	2.18	8452.2
5	10	0.570	7.78	1.72×10^7	1.56	1.72×10^6
10	10	0.694	23.09	1.61×10^9	2.31	1.61×10^8

Figure S 4 Antitumor responses to combined radiotherapy and chemotherapy in C-33A spheroids. C-33A spheroids were maintained in ultra-low attachment plates for 24 h, irradiated with a total dose of 2.5 Gy, 5 Gy or 10 Gy and immediately treated for 48 h with P8-D6, cisplatin or PBS. (A top) After treatment, C-33A spheroids were live/dead cell co-stained with PI (red), calcein-AM (green) and imaged by fluorescence microscopy; scale bars, 500 µm. (A bottom) Scanning electron microscope images were taken of spheroids treated with radio-chemotherapy; scale bars, 50µm. (B) Viability and caspase activity of C-33A spheroids were measured after treatment, (relative luminescence units, RLU). (C, E) Combination index (CI) for combining radiotherapy with P8-D6 (C) or cisplatin (E) was determined using C-33A spheroids. CI values was calculated by CompuSyn software using the fraction affected (fa)-value. The fa-value represents the fraction of cell viability affected by therapy. fa color scalar (0 (white)-1 (light brown)). Combinations were considered synergistic when CIs were below 1.0. (D, F) The monotherapy column defines the concentrations that are

needed in monotherapy to affect a certain fraction of cells by therapy with P8-D6 (D) or cisplatin (F) and radiotherapy. The increasing blue color intensity classifies the concentration required to achieve the same effect. The more treatment required, the more intense the blue color. DRI values represents the order of magnitude (fold) of dose reduction in combination setting compared with each drug alone.

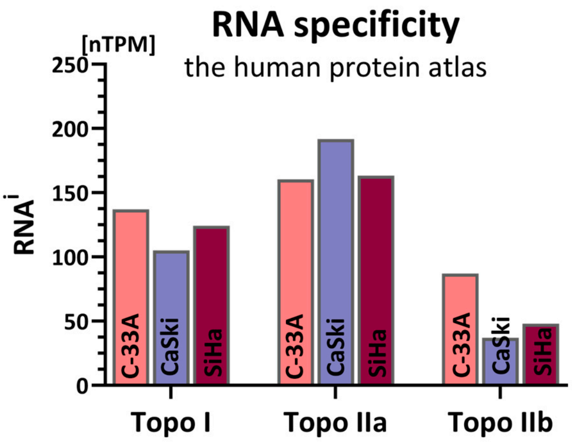

Figure S 5 mRNA expression of cervical cell lines by humane protein atlas [1].
RNA expression profile of DNA topoisomerase I (Topo I), DNA topoisomerase II alpha (Topo IIa) and DNA topoisomerase II beta (Topo IIb) in human cervical cancer cell lines. Data from the Human Protein Atlas (proteinatlas.org).

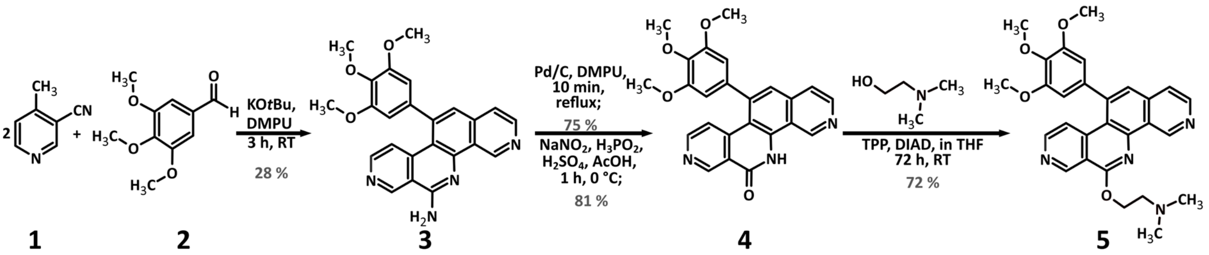

Figure S 6. Synthesis of P8-D6 (5).
KOtBu (potassium tert-butoxide), Pd/C (palladium on carbon), $NaNO_2$ (sodium nitrite) H_3PO_2 (hypophosphorous acid), H_2SO_4 (sulfuric acid), AcOH (acetic acid), TPP (triphenylphosphine), DIAD (diisopropyl azodicarboxylate) [2].

1. Thul, P.J.; Åkesson, L.; Wiking, M.; Mahdessian, D.; Geladaki, A.; Ait Blal, H.; Alm, T.; Asplund, A.; Björk, L.; Breckels, L.M.; et al. A subcellular map of the human proteome. *Science* **2017**, *356*, doi:10.1126/science.aal3321.
2. Meier, C.; Steinhauer, T.N.; Koczian, F.; Plitzko, B.; Jarolim, K.; Girreser, U.; Braig, S.; Marko, D.; Vollmar, A.M.; Clement, B. A Dual Topoisomerase Inhibitor of Intense Pro-Apoptotic and Antileukemic Nature for Cancer Treatment. *ChemMedChem* **2017**, *12*, 347–352, doi:10.1002/cmdc.201700026.
