# Supplementary figures and images for: Dual Topoisomerase Inhibitor Is Highly Potent and Improves Antitumor Response to Radiotherapy in Cervical Carcinoma
Source: Int J Mol Sci. 2025 Mar 21;26(7):2829. doi: 10.3390/ijms26072829 (PMC11988843; doi:10.3390/ijms26072829)

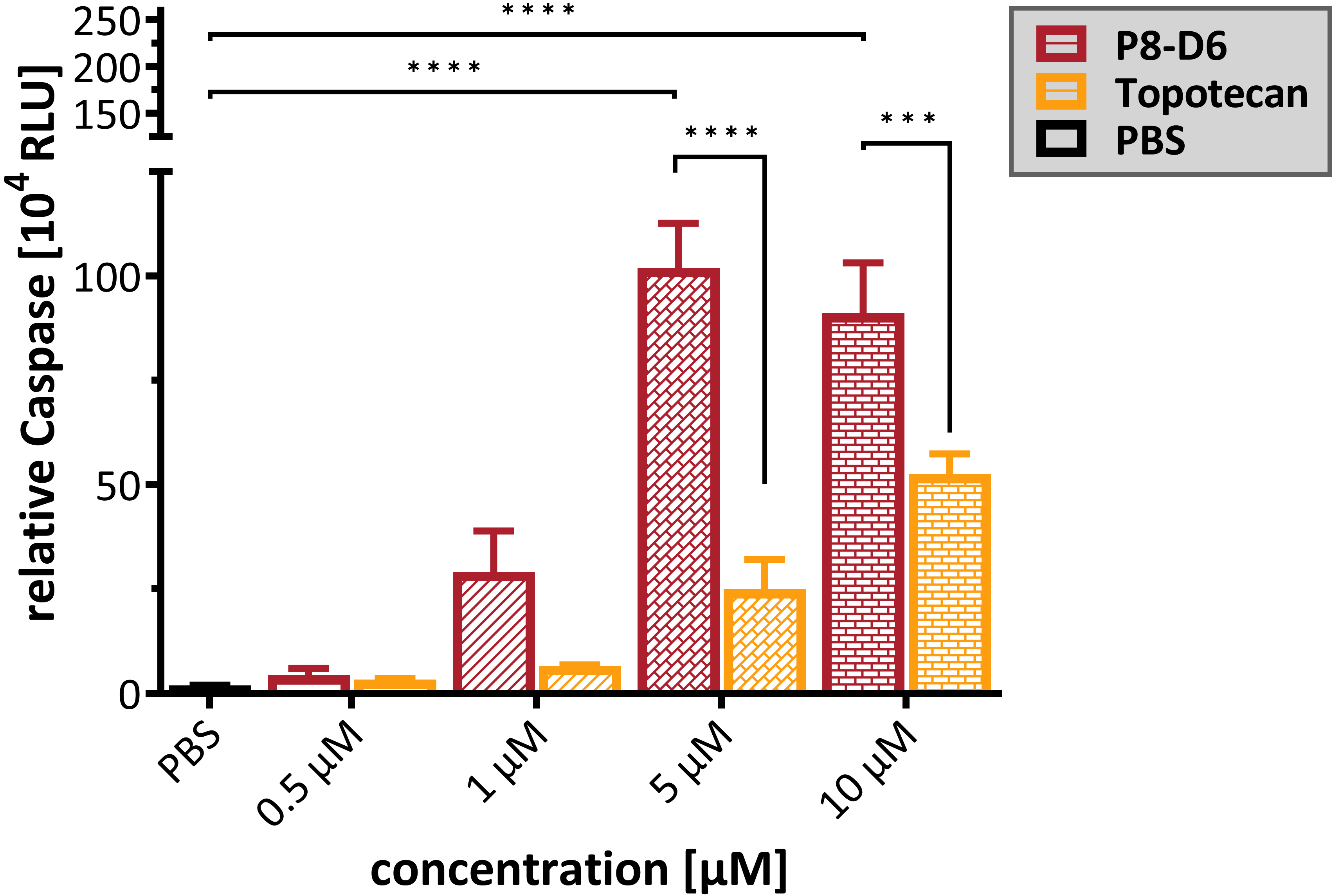

Supplement: Supplementary file 1 [file ijms-26-02829-s001.zip › Supplementary Flies/Figure S 1.png]

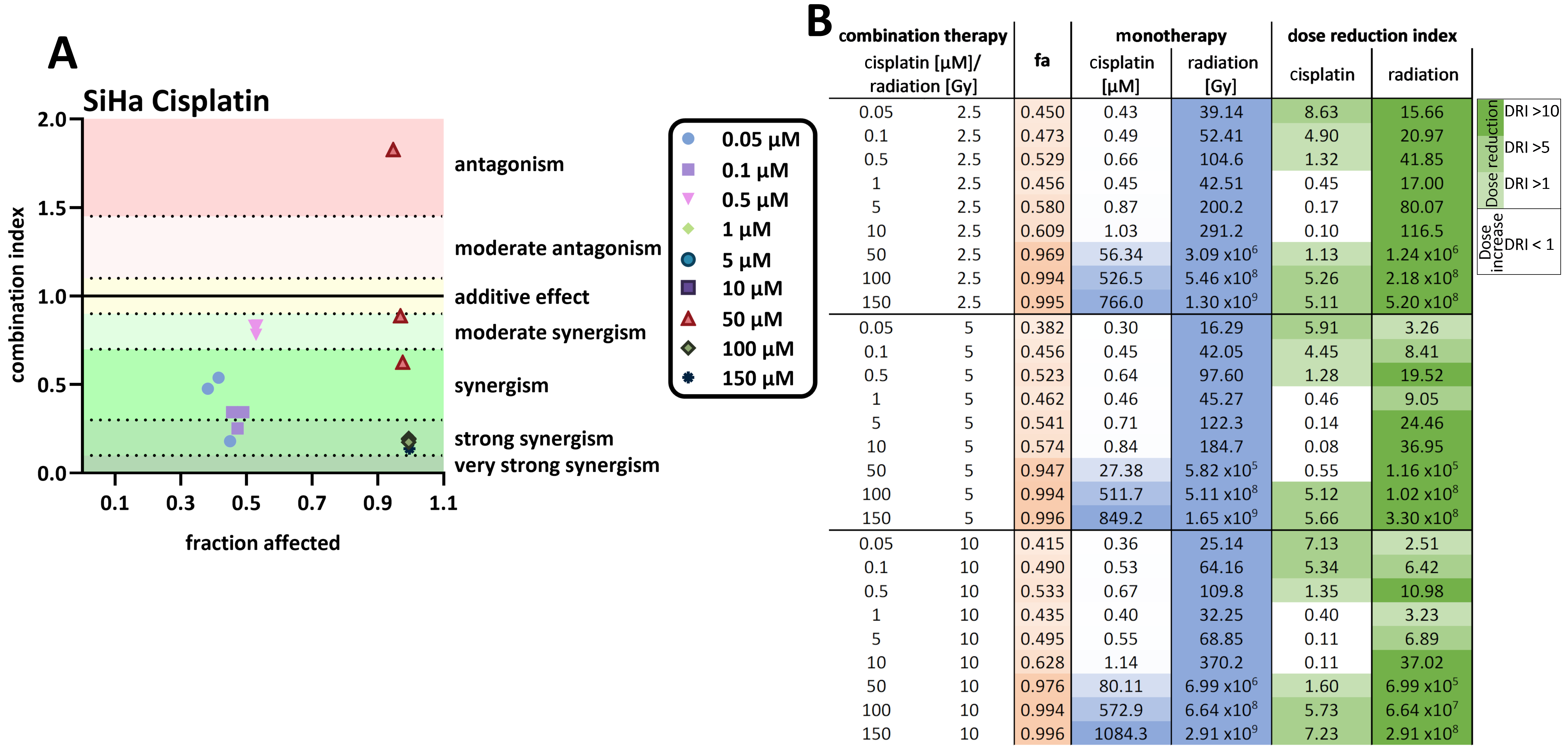

Supplement: Supplementary file 1 [file ijms-26-02829-s001.zip › Supplementary Flies/Figure S 2.png]

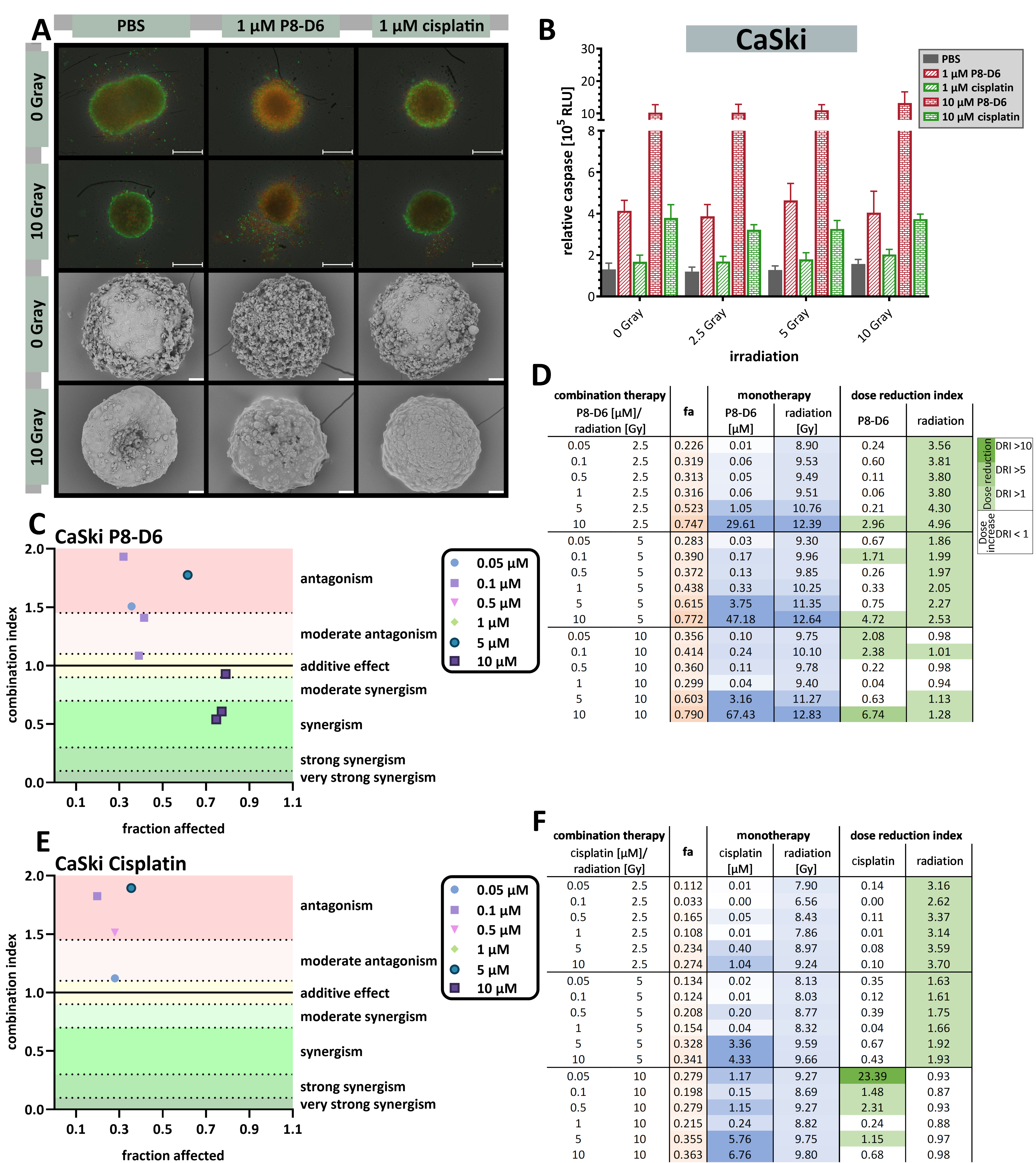

Supplement: Supplementary file 1 [file ijms-26-02829-s001.zip › Supplementary Flies/Figure S 3.png]

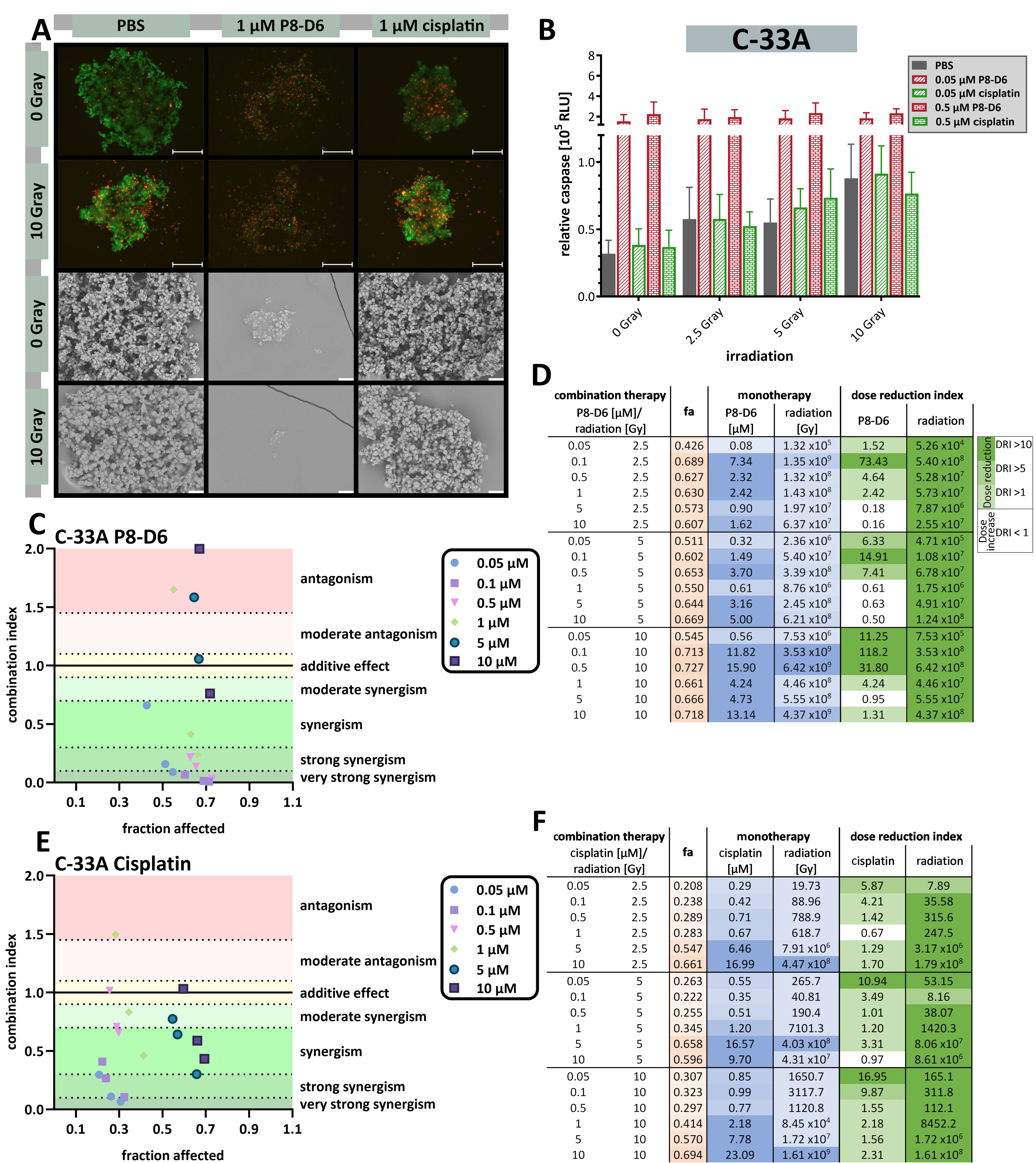

Supplement: Supplementary file 1 [file ijms-26-02829-s001.zip › Supplementary Flies/Figure S 4.png]

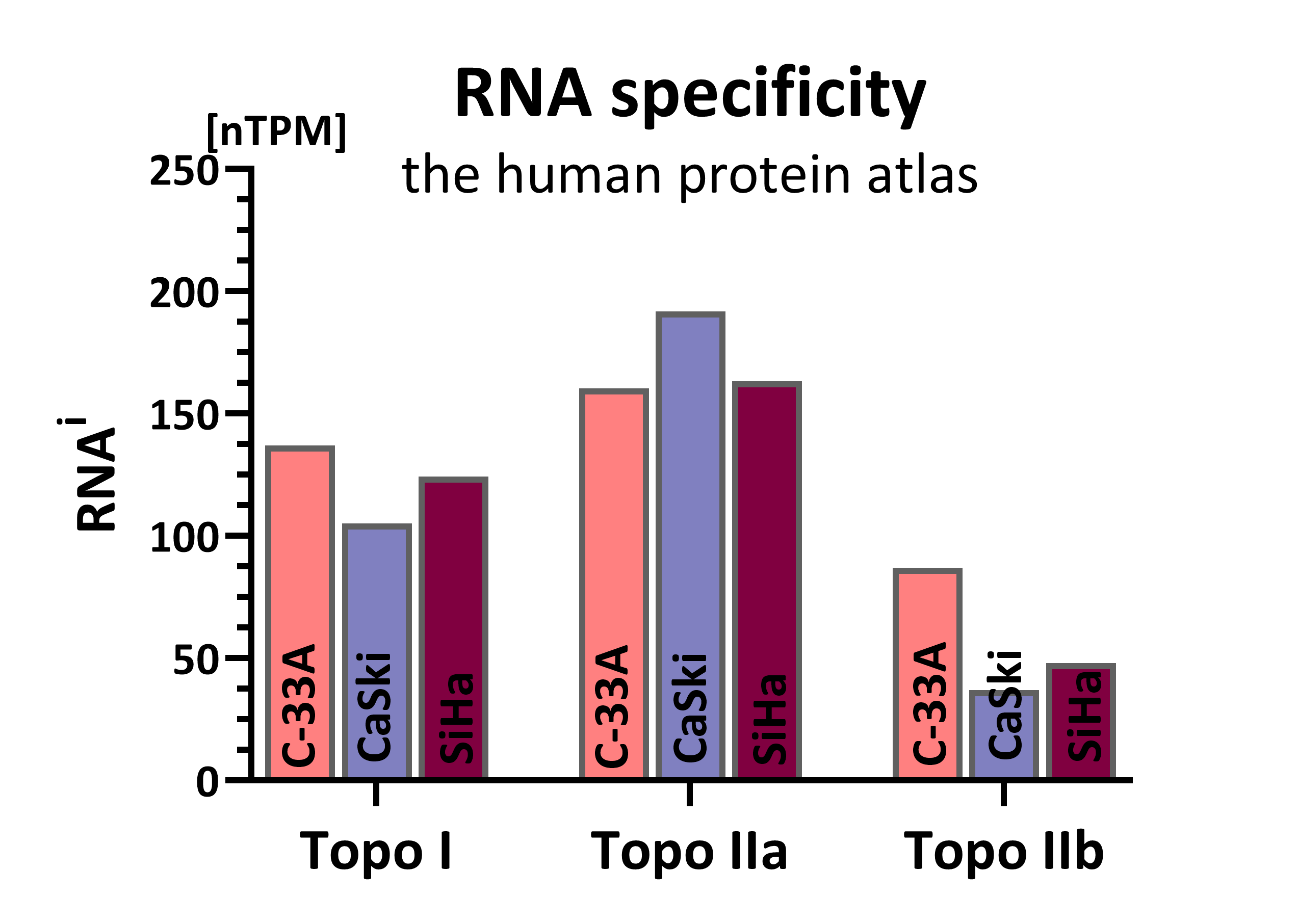

Supplement: Supplementary file 1 [file ijms-26-02829-s001.zip › Supplementary Flies/Figure S 5.png]

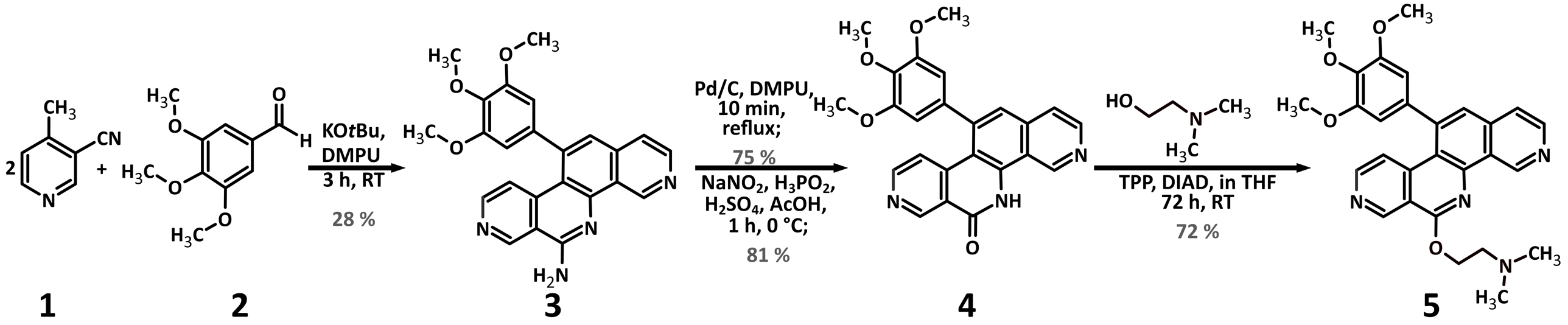

Supplement: Supplementary file 1 [file ijms-26-02829-s001.zip › Supplementary Flies/Figure S6.png]
